# Hydrogenation Properties of TiFe Doped with Zirconium

**DOI:** 10.3390/ma8115423

**Published:** 2015-11-20

**Authors:** Catherine Gosselin, Jacques Huot

**Affiliations:** Hydrogen Research Institute, Université du Québec à Trois-Rivières, 3351 des Forges, Trois-Rivières, QC G9A 5H7, Canada; Catherine.gosselin1@uqtr.ca

**Keywords:** TiFe alloy, hydrogen storage, metal hydrides, kinetics, air exposure

## Abstract

The goal of this study was to optimize the activation behaviour of hydrogen storage alloy TiFe. We found that the addition of a small amount of Zr in TiFe alloy greatly reduces the hydrogenation activation time. Two different procedural synthesis methods were applied: co-melt, where the TiFe was melted and afterward re-melted with the addition of Zr, and single-melt, where Ti, Fe and Zr were melted together in one single operation. The co-melted sample absorbed hydrogen at its maximum capacity in less than three hours without any pre-treatment. The single-melted alloy absorbed its maximum capacity in less than seven hours, also without pre-treatment. The reason for discrepancies between co-melt and single-melt alloys was found to be the different microstructure. The effect of air exposure was also investigated. We found that the air-exposed samples had the same maximum capacity as the argon protected samples but with a slightly longer incubation time, which is probably due to the presence of a dense surface oxide layer. Scanning electron microscopy revealed the presence of a rich Zr intergranular phase in the TiFe matrix, which is responsible for the enhanced hydrogenation properties of these Zr-doped TiFe alloys.

## 1. Introduction

Solid state hydrogen storage in metal hydrides is a promising form of energy storage for stationary machines, backup power, and for heavy duty vehicles. In these applications, the system safety, low maintenance, compactness, and operating costs far exceed the criteria of high gravimetric hydrogen capacity. One important aspect of this form of storage is the potential for reversibility at room temperature, needed for most commercial and industrial applications [1].

Among the different metal hydrides, TiFe is one of the low cost intermetallic compounds that operate near room temperature (RT) and under a mild pressure environment [2]. However, TiFe alloy has poor activation characteristics, needing time, high pressure, and high temperature to achieve full hydrogenation [3]. This is a major concern for commercial and industrial applications. In order to overcome this problem, different approaches have been investigated. They include pulse current-assisted reaction [4], ball milling [3,5], plastic deformations [6], and utilizing clusters [7].

Another approach uses element substitution for Fe or Ti with transition metals (TMs), including Mn, Cr, Ni and Zr [8,9,10,11,12]. The partial substitution of Fe with TMs improves the activation process by reducing the time, pressure, and heat needed [13]. 

We have recently exposed that the addition of a small amount of Zr_7_Ni_10_ alloy to TiFe drastically improves the activation process. In this study, we found that the enhancement was due to the two-phase microstructure consisting of an intergranular phase and an iron-titanium matrix. As the intergranular phase was found to contain most of the zirconium, we wanted to test the hypothesis that zirconium is the main element responsible for the activation improvement [14]. Additionally, it was reported by Nagai *et al.* [13] that substituting iron with zirconium greatly improved the activation kinetics and hydrogen capacity. In this paper we report the investigation study of the use of Zr to facilitate the activation behaviour of TiFe alloy. Therefore, the amount of zirconium added was much less than in Nagai’s investigation. Nagai investigated the compositions TiFe_1-y_Zr_y_ where y = 0.1, 0.2, and 0.3, which translate to an amount of zirconium from 8 to 23 wt.%. In our case, we wanted to investigate the effect of adding a much smaller amount of Zr than in Reference [13], in the hopes of enhancing activation behaviours of TiFe-based alloys. The compositions studied were TiFe + xZr, where x = 0, 2, and 4 wt.%. In the present investigation, the effect of the synthesis method and Zr proportion on activation behaviour was measured. The study of microstructure and element distribution in the alloy gave some indication of the possible mechanism responsible for the faster activation process. 

## 2. Results and Discussion

### 2.1. Activation Process

**Figure 1:**
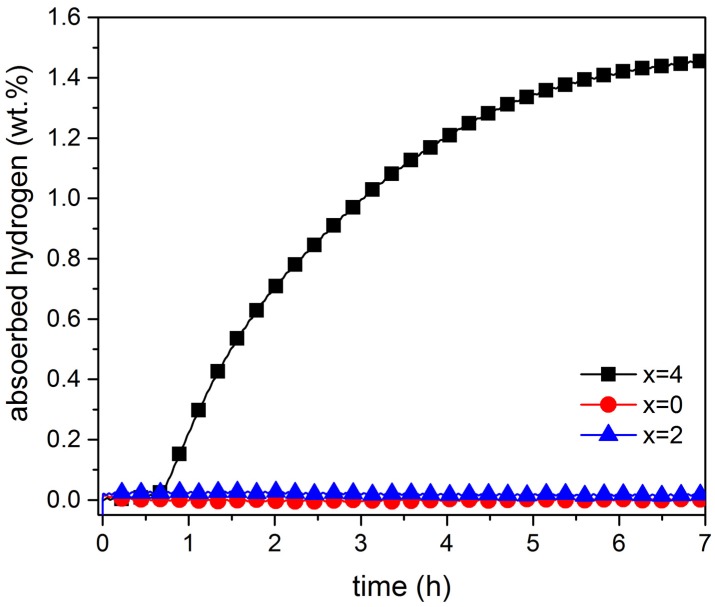
Activation curves at room temperature under 4500 kPa of hydrogen of single-melt TiFe + x wt.% Zr for x = 0, 2, 4.

The kinetics of the first hydrogenation, also called activation, of single melt TiFe + x wt.% Zr (x = 0, 2, 4) alloys are shown in Figure 1. At room temperature, and under a hydrogen pressure of 4500 kPa, the samples with 0% and 2% did not absorb hydrogen, even after seven hours of exposure.

Addition of 4 wt.% zirconium yielded a significant improvement in activation time. After an incubation time of less than one hour, the alloy started to absorb hydrogen, reaching a maximum storage capacity of 1.45 wt.%.

**Figure 2:**
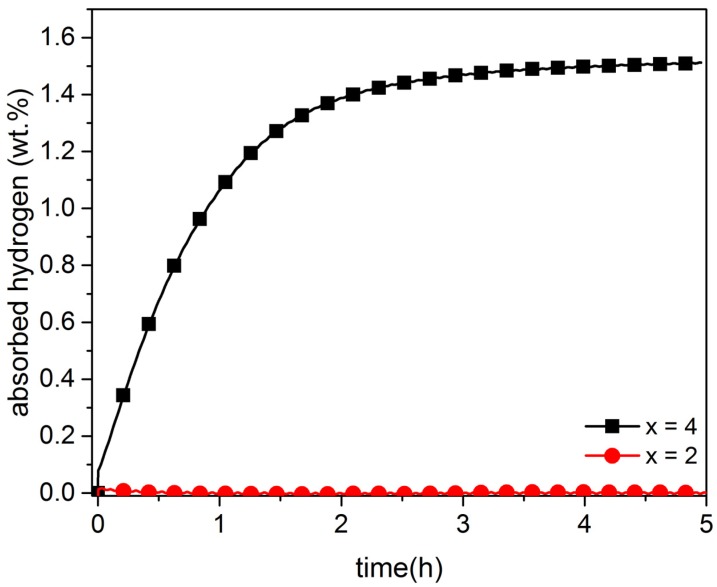
Activation curves at room temperature under 4500 kPa of hydrogen of co-melt TiFe + x wt.% Zr for x = 2, 4.

Figure 2 shows the rate of absorption of hydrogen for co-melt TiFe with a content of 2 and 4 wt.% of zirconium. Under these conditions, and similar to the single-melt sample, the samples with 2 wt.% did not absorb hydrogen, even after five hours of exposure. The sample with 4 wt.% zirconium shows no incubation time and readily absorbs hydrogen. In this case, the maximum storage capacity of 1.59 wt.% was reached after four hours.

Thus, it is clear that the addition of 4 wt.% of zirconium drastically improves the first hydrogenation (activation) kinetics for both single and co-melt alloys. The main difference between them is that the co-melt alloy does not exhibit an incubation time.

#### Air Exposure Effect

**Figure 3:**
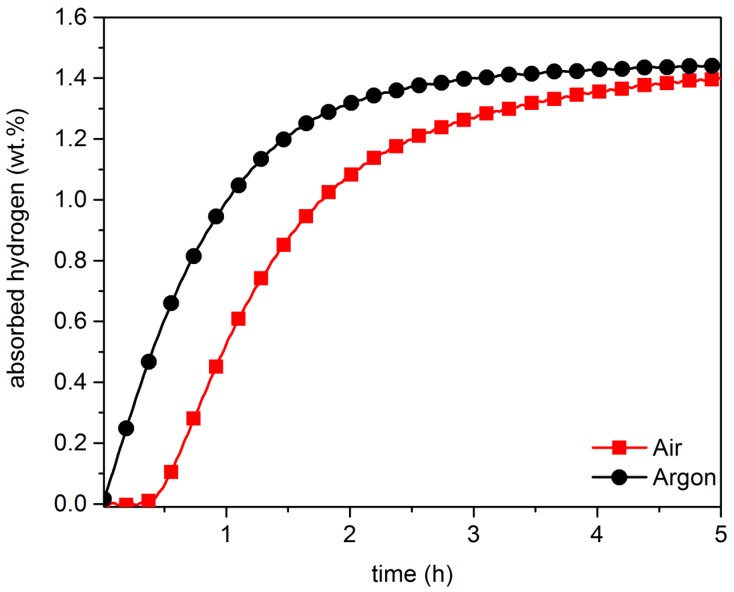
Activation curves of co-melt of TiFe + 4 wt.% Zr under Argon and under air, at room temperature, with 4500 kPa of hydrogen.

**Figure 4:**
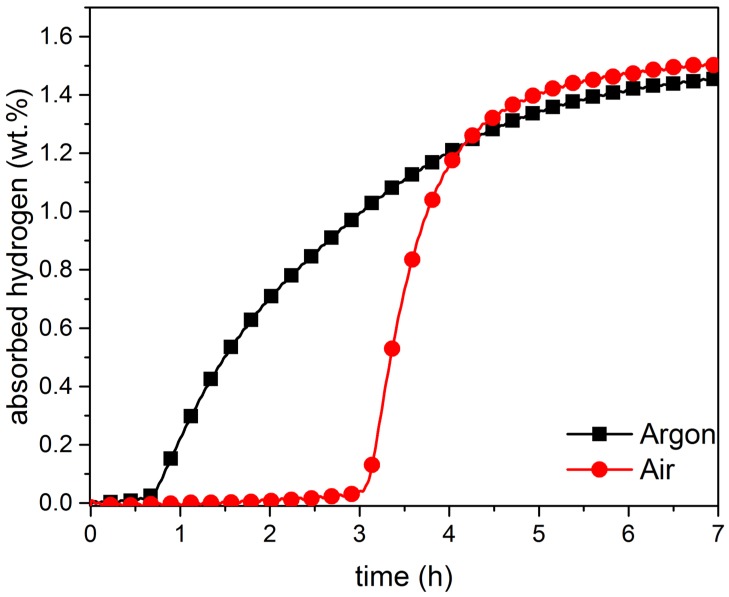
Activation curves of single-melt of TiFe + 4 wt.% Zr, under argon and under air, at room temperature. Applied hydrogen pressure is 4500 kPa.

Being able to handle alloys in air is a critical requirement for large-scale production. For this reason, we tested the effect of air exposure by crushing the alloys in air. Figure 3 and Figure 4, respectively, show the activation curve of co-melt and single-melt TiFe + 4 wt.% Zr samples handled in air and in argon.

For both single-melt and co-melt samples, handling in air result in degradation of activation behaviour. In the case of co-melt (Figure 3), a short incubation time was present, but, afterwards, the absorption kinetics were very similar to the argon exposed sample.

The single-melt sample handled in air (Figure 4) presented a much longer incubation time than its argon handled counterpart, but the hydrogenation kinetics after the incubation time were much faster. This led to the unexpected result that full capacity was reached faster in the case of air handled sample. Clearly, the incubation time is linked to the fact that the oxide layers, caused by the exposure to air, prevent the penetration of hydrogen in the alloys. However, presence of some level of oxides may, in fact, speed up the intrinsic kinetics of hydrogenation. 

It should be pointed out that the air-exposed alloy did not suffer a significant loss of hydrogen capacity. This could be explained by the fact that, as explained in the Experimental Section, the material was crushed by hand using a mortar and pestle. After crushing, the material is composed of very coarse grains (particles more than 400 μm in diameter), thus, the surface/volume ratio was still very small. This means that, even if the surfaces of particles are oxidized, the volume of oxidized material is still very small. When crushed in air, all surfaces are oxidized and this is the reason for the long incubation time. However, when hydrogen could penetrate this oxide surface, the increases of volume of the hydride phase fracture the material and expose fresh surfaces. This is the reason for the fast kinetics after the incubation time.

### 2.2. Structure

**Figure 5:**
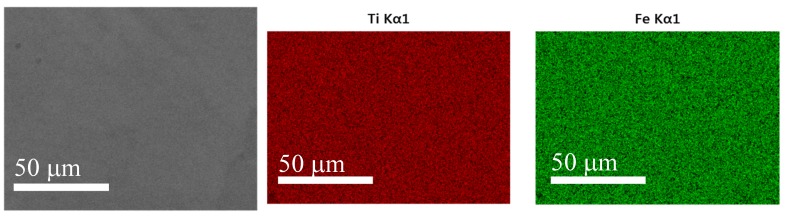
Secondary electron micrograph and EDX mapping of TiFe alloy.

From the activation measurement reported above, it is clear that activation depends, not only on the chemistry, but also on the synthesis method. This led us to study the alloy’s microstructure and element distribution. Figure 5 shows the micrograph and element mapping of single-melt TiFe alloy. It is clear that the elements are evenly distributed and, at this scale, there is no intergranular phase. It should be pointed out that the alloy was not etched after polishing. This is the reason why the micrograph appears structureless. The bulk abundance in atomic % of Ti and Fe, as measured by EDX, was, respectively, 50.6 ± 0.1 and 49.4 ± 0.1, which is very close to the nominal composition.

**Figure 6:**
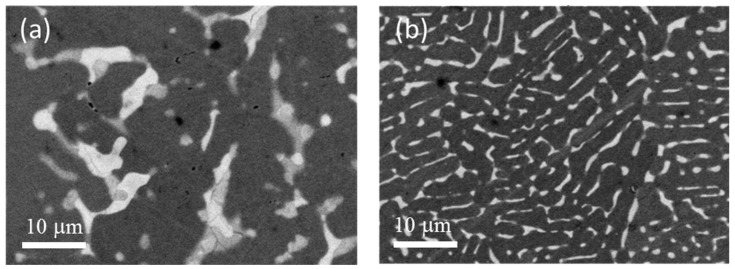
Secondary electron micrographs of (**a**) co-melt and (**b**) single melt TiFe + 4 wt.% Zr alloys.

Figure 6 shows the micrographs of single-melt and co-melt of TiFe + 4 wt.% Zr alloy. It is clear that the microstructure of these two alloys is totally different. The single-melt alloy has a much smaller and more evenly distributed intergranular phase. Table 1 shows that the bulk abundance of each alloy, as determined by EDX, agrees with the nominal abundance.

**Table 1:** Bulk atomic abundance as measured by EDX of TiFe + 4 wt.% Zr single-melt and co-melt alloys. Error on each composition is indicated in parentheses.

TiFe + 4 wt.% Zr			
Alloys	Atomic %		
	Ti	Fe	Zr
Nominal composition	47.85	47.85	4.30
Co-melt	50.7 (0.2)	45.8 (0.2)	3.5 (0.2)
Single-melt	49.0 (0.2)	48.3 (0.2)	2.7 (0.2)

**Figure 7:**
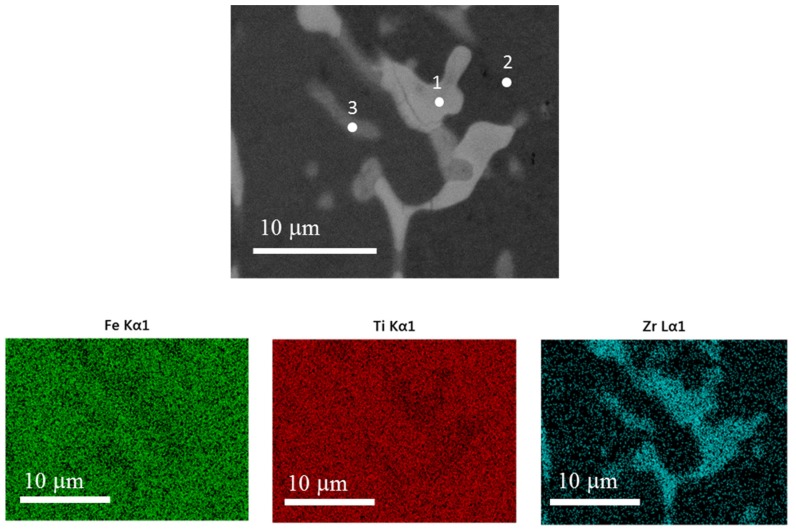
Secondary electron micrograph and EDX mapping of TiFe + 4 wt.% Zr co-melt alloy. Numbers in the first image indicate specific points reported in Table 2.

Figure 7 presents a higher magnification micrograph of the co-melt sample, along with element mapping. It is clear that zirconium is mainly located in the intergranular phase. The atomic abundances at specific points indicated in Figure 7 are listed in Table 2.

Close inspection of Table 2 indicates that a small amount of zirconium diffused into the matrix. The two intergranular regions studied seem to have a composition close to (Ti_1-y_Zr_y_)_2_Fe. This composition is the one found by Nagai *et al.* when they substituted iron with zirconium [13]. In their paper, they concluded that it was the dispersion of this (Ti_1-y_Zr_y_)_2_Fe phase in TiFe alloy that significantly accelerated the activation process. The present results agree with that conclusion.

**Table 2:** Atomic abundance at specific points as measured by EDX of TiFe + 4 wt.% Zr co-melt alloy. Error on each number is indicated in parentheses.

TiFe + 4 wt.% Zr Co-Melt			
Localization	Atomic %		
	Ti	Fe	Zr
Point 1 (intergranular phase)	42.2 (0.3)	39.2 (0.3)	18.6 (0.2)
Point 2 (matrix)	50.7 (0.3)	48.6 (0.3)	0.8 (0.2)
Point 3 (intergranular phase)	51.6 (0.3)	34.8 (0.2)	13.6 (0.2)

**Figure 8:**
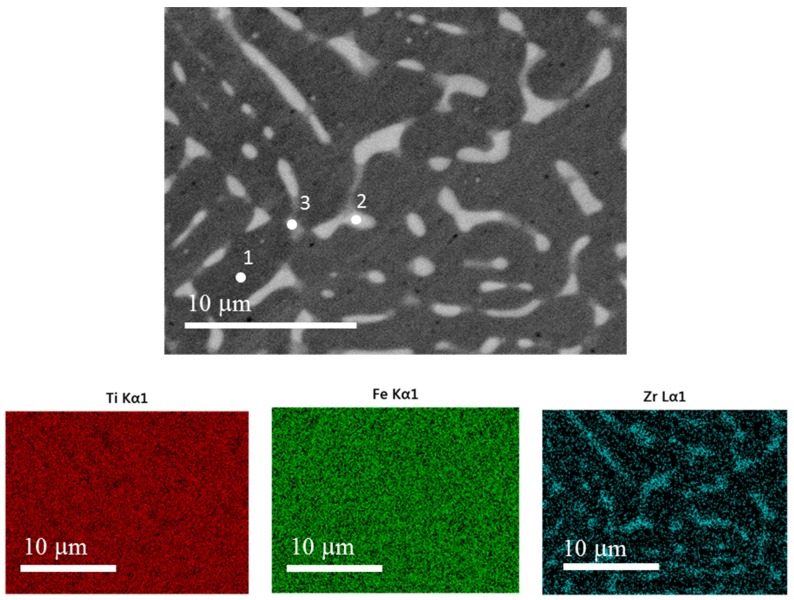
Secondary electron micrograph and EDX mapping of TiFe + 4 wt.% Zr single-melt alloy. Numbers in the first image indicate specific points reported in Table 3.

The single-melt alloy presents a different microstructure, and element mapping as can be seen in Figure 8. With respect to the co-melt alloy, the zirconium is mainly located in the intergranular phase. Table 3 lists the atomic abundance at the specific points marked in Figure 8.

Table 3 indicates that, as in the case of co-melt alloy, only a small of zirconium diffused into the matrix. Moreover, the amount of Zr in the solid solution in TiFe is the same as the co-melt alloy. Zirconium is mainly present in the intergranular phase, as was seen for the co-melt alloy, but the elemental composition of the intergranular phase is quite different. Here, the stoichiometry is not of the type (Ti_1-y_Zr_y_)_2_Fe, but closer to (Ti_1-y_Zr_y_)Fe or (Fe_1-y_Zr_y_)_2_Ti. Thus, the distribution and chemistry of the intergranular phase is completely different for the co-melt and single-melt alloys. This may explain the discrepancy in activation behaviour between single and co-melt alloys.

**Table 3:** Atomic abundance at specific points as measured by EDX of TiFe + 4 wt.% Zr single-melt alloy. Error of each number is indicated in parentheses.

TiFe + 4 wt.% Zr Single-Melt			
Localization	Atomic %		
	Ti	Fe	Zr
Point 1 (matrix)	50.1 (0.3)	49.1 (0.2)	0.8 (0.2)
Point 2 (Intergranular)	39.6 (0.3)	47.9 (0.3)	12.5 (0.2)
Point 3 (intergranular)	49.1 (0.3)	41.6 (0.3)	9.3 (0.2)

The hydrogen capacities measured are smaller than the nominal value of 1.86 wt.% for a fully hydride TiFeH_2_. At this moment we do not have a clear explanation for this fact. However, two possible explanations could be put forward. First, the capacities measured here are for the first cycle (activation). It is well known that, for many metal hydrides, several hydrogenation/dehydrogenation cycles have to be performed before reaching full capacities. In the present case, we have shown that the addition of Zr drastically improves the activation, but it may still be necessary to perform a few more cycles in order to reach full capacity. This is the subject of an ongoing experiment. Secondly, the presence of the intergranular phase may potentially reduce the total reversible capacity. As the intergranular phase seems to form a hydride more easily than the main TiFe phase, we suspect that this phase is much more stable and, thus, may possibly stay in a hydride state during a hydrogenation/dehydrogenation cycle of the main TiFe phase. This is the reason why we did not investigate higher Zr contents. The goal of this study was to find a way to get a faster activation without changing the thermodynamics and capacity of the TiFe alloy. Therefore, we just wanted to add the minimum amount of Zr to improve the activation kinetics. 

## 3. Experimental Section

Commercial Fe (99.9%), Ti sponge (99.9%), and Zr sponge (99.5%) were all purchased from Alfa Aesar (City, Country) and used without further purification. Syntheses of TiFe alloy and TiFe-Zr were done using arc melting. One method of preparation involved separately melting TiFe alloy, and, thereafter, adding Zr and re-melting them together. This process is called co-melting. Alloys were synthesized using the single-melt method, where all the elements are melted together. For both methods, melting was done using an arc melting apparatus working at 240 volts (V) and 60 amperes (A). For each melting, the pellet was turned over 3 times and re-melted in order to ensure homogeneity. The pellet was then hand crushed using a steel mortar and pestle under Argon atmosphere or in air. The material was filled in a reactor cell and kept under vacuum for 1 h at room temperature before exposing it to hydrogen. The hydrogen sorption properties were measured using a home-made Sievert type apparatus. The measurements were done at room temperature (RT) under a hydrogen pressure of 4500 kPa for absorption and 100 kPa for desorption. Morphological studies and chemical analysis were made on a JEOL JSM-5500 scanning electron microscope. 

## 4. Conclusions

In this study, the effect of the zirconium on TiFe alloy was investigated from the perspective of improving the activation of this system. The addition of 4 wt.% zirconium is the minimum threshold in order to activate the compound without any pre-treatment. The synthesized alloys were processed under argon and in the air prior to activation. Compared to the alloy prepared under argon, the air-exposed alloy had a similar rate of absorption and maximum capacity. The only difference was the incubation time, which was longer when the alloy was processed in air. The improvement of activation of the alloy is caused by the specific microstructure and chemical composition of the intergranular phase. An intergranular phase of stoichiometry (Ti_1-y_Zr_y_)_2_Fe seems to be more effective for activation. The whole process remained simple and inexpensive. The aim of this investigation was to optimize the activation behaviour of TiFe to make a simple and cost-effective material that would be efficient in storing hydrogen under mild pressure at room temperature. It should be pointed out that we did not fine-tune the amount of necessary zirconium for the following reason: in this investigation, the alloys were synthesized by arc melting. However, in industry, the alloy will be made using a totally different casting technique. As the microstructure heavily depends on the casting parameters, in this study we wanted to set the general chemical composition that facilitates activation. In future work we will use induction melting to cast the alloys. Then, it will be more appropriate to fine-tune the chemical composition and study the impact of microstructure.
